# The role of the industrial psychologist in managing the psychological impact of COVID-19 in the workplace

**DOI:** 10.3389/fpsyg.2022.920894

**Published:** 2022-08-01

**Authors:** Thapelo Sendry Moralo, Lené Ilyna Graupner

**Affiliations:** WorkWell Research Unit, Faculty of Economic and Management Sciences, School of Industrial Psychology and Human Resource Management, North-West University, Potchefstroom, South Africa

**Keywords:** COVID-19, psychological impact, mental health, industrial psychology, workplace counseling

## Abstract

Recently, the world experienced dramatic changes due to the onset of the COVID-19 pandemic. Working remotely led to employees feeling isolated and experiencing fatigue and depression. The responsibility of addressing the psychological wellbeing of employees lies with industrial psychology practitioners. They support line management by counseling employees experiencing social and psychological problems. The objective of the present study was to explore the role of the industrial psychology practitioner in managing the psychological impact of COVID-19 on employees. Using a homogeneous sampling technique, a qualitative research design was employed based on social constructivism. Semi-structured interviews and a qualitative survey were utilized to gather the data from industrial psychology practitioners (*n* = 22) registered as psychologists and interns. Thematic analysis was employed to analyze the data. Most participants believed that the onset of COVID-19 led to accelerated change in the workplace. The findings suggest that an industrial psychology practitioner’s role in the changing world of work enables organizations to be prepared for the changes by providing multi-level interventions. Recommendations are made to organizations to implement interventions to facilitate support for employees in their attempt to deal with the psychological impact of COVID-19 on employees.

## Introduction

The workplace has a vital role in helping employees deal with social and psychological concerns (Bergh, 2021). These typically include navigating through the ever-changing world of work, such as the dramatic changes the world experienced due to the COVID-19 pandemic. The pandemic led to strict lockdown measures and social isolation worldwide, and employees working remotely from the workplace while managing their home lives. Studies show that people reported feeling anxious and stressed during the pandemic due to the threat of falling ill, job insecurity, isolation and uncertainty for the future, to name a few (Giorgi et al., 2020; Qiu et al., 2020). However, COVID-19 also brought about embracing new insights and capacities, thereby completely rethinking the future world of work (Volini et al., 2021). In this regard, the role of the industrial and work psychology practitioner becomes evident. The responsibility for addressing psychological issues within South African organizations lies with industrial psychology practitioners (Health Professions Council of South Africa [HPCSA], 2019). The industrial psychology practitioner is responsible for diagnosing workplace-related psychopathology and applying skills to identify further treatment needs and psychological interventions (Health Professions Council of South Africa [HPCSA], 2019). The practitioner can refer employees to other specialized professionals and work with them to ensure that the employee is reasonably accommodated and integrated into the workplace (Health Professions Council of South Africa [HPCSA], 2019). The role of the industrial psychology professional is imperative to provide support to employees to adjust to the changes COVID-19 brought to the workplace. From the aforementioned, the following research objectives are presented.

To determine the role of the industrial psychology practitioner in the changing world of work;
To establish the role of the industrial psychology practitioner as a workplace counselor to manage the impact of COVID-19 in the workplace.

In response to the COVID-19 pandemic, governments worldwide were forced to impose regulations such as lockdowns and social distancing (Greyling et al., 2021). During the onset of this study, most employees in South Africa have already been working from home for several months. Most companies used technology for employees to do their work using new virtual ways of working, such as virtual communication platforms such as Zoom meetings and Microsoft Team meetings, among others (Williams, 2021). Working from home, enabled by advanced technologies, caused problems for some employees as it faded the line between working time and family time (Trougakos et al., 2020). Greenwood and Anas (2021) state that employers were only beginning to recognize the prevalence of mental health issues at work in 2019 when COVID-19 brought new mental health challenges. The global pandemic declared by the World Health Organization (WHO) in 2020 exacerbated existing work-related problems and how mental health-related issues were managed (Agba et al., 2020). Giorgi et al. (2020) state that several workplace factors have been identified as having the potential to increase or moderate the impact of COVID-19 on workers’ mental health. During the pandemic, work-related stress and a lack of job support were intrinsically at a high risk of influencing the wellbeing of employees (Giorgi et al., 2020).

Similarly, Qiu et al. (2020) report that the pandemic caused increased anxiety and psychological distress among people. According to Devi et al. (2019), stress is defined as a state of disharmony leading to a stress response in the body. Prolonged exposure to stress leads to health-related consequences on productivity and quality of life (Devi et al., 2019). The Job Demands-Resources model (Demerouti et al., 2001) states that when the availability of job resources is depleted, a person could withdraw or disengage from work. High job demands lead to exhaustion and could eventually cause ill health. According to Devi et al. (2019), prolonged exposure to stress could lead to post-traumatic stress disorder (PTSD), which usually occurs following a life-threatening event, such as the COVID-19 pandemic. Several publications report on employees’ symptoms of post-traumatic stress caused by the pandemic (Restauri and Sheridan, 2020; Rock, 2022). There are various interventions organizations can implement in order to support employees to recover from the psychological trauma and distress caused by the pandemic. According to Giorgi et al. (2020), managing the psychological effect of COVID-19 would include multiple organizational and work-related interventions, such as remote working (smart working), leadership support and implementation of safety protocols. Giorgi et al. (2020) further report that a proper strategy to address employee burnout is to apply coaching psychology principles. Facilitating a development process and establishing ways for employees to improve their resources could assist in addressing COVID-19-related challenges. Identifying at-risk employees and training in psychological first aid during disasters could prevent added mental health problems (Naidu, 2020; Qiu et al., 2020). Currently, it seems that the world is in a post-pandemic phase. Early this year most countries have lifted lockdown restrictions, and some organizations require employees to return to work. Recent studies report on managing return-to-work policies since this significantly affects employees (Giorgi et al., 2020; Boissé, 2021; Cohen, 2021).

In this regard, the industrial psychology practitioner’s role becomes crucial. The Health Professions Council of South Africa (Health Professions Council of South Africa [HPCSA], 2019) indicates that an industrial psychologist should develop and apply interventions based on the principles of psychology to address individual, group and organizational wellbeing. A subfield in industrial psychology relating to work-related wellbeing, occupational health psychology focuses on the impact of occupational stressors on employees’ psychological health and includes interventions designed to improve employee health (Bergh, 2021). Bal et al. (2019) state that industrial psychology practitioners are responsible for ensuring the wellbeing of the employee, as this task forms the cornerstone of their profession. The industrial psychologist uses psychological models to alter behavior in organizations in order to improve work (Schultz et al., 2020) and resolve issues at work (Jex and Britt, 2014). Schultz et al. (2020) note that industrial psychology practitioners’ responsibilities include providing psychological interventions to businesses, such as workplace counseling, to address psychological problems. Basically, through workplace counseling, an industrial psychology practitioner assists workers to function optimally at work. From an industrial psychology perspective, workplace counseling focuses on facilitating workers’ personal development while also addressing stress-related problems. From this perspective, this study aimed to explore the significant role of the industrial psychologist in managing the psychological impact of COVID-19 in the workplace.

## Materials and methods

A qualitative descriptive strategy was used in the study to explore the narratives of the participants, industrial psychology practitioners registered as psychologists and interns (we refer to the collective term of industrial psychology practitioner to include both psychologists and interns). The inclusion criterion for the participants consisted of being registered with the Health Professions Council of South Africa (HPCSA) as an intern- psychologist or psychologist under the industrial psychology category. It was preferred that the practitioners’ job functions include rendering workplace counseling within a South African organization. The participants were expected to understand and communicate in English. After approval for the study was received from the ethics committee from the faculty, the list of registered industrial psychology practitioners from the iRegister of the HPCSA was obtained. The participants were contacted, and an overview of the study was presented. Provided they agreed to participate in the study, an informed consent form and a brief demographics questionnaire were completed.

The sample comprised 17 females (77%) and five males (22%) mainly between 33 and 41 years of age (46%). 36% of the participants were between 23 and 32 years of age, and four (18%) participants between 42 and 51 years of age. The participants were equally Afrikaans-speaking and English-speaking at 36%, respectively, while 14% were isiZulu-speaking, 9% were Setswana-speaking, and 5% were Sesotho-speaking. 18% Asian represented the sample population, 31% black, 9% colored and 9% white. Lastly, 59% of the participants were registered with the HPCSA as psychologists in the category *industrial*, and 41% were registered with the HPCSA as *intern psychologists* in the same category.

Semi-structured interviews and a qualitative survey were used to gather data. Since the country was under lockdown restrictions during 2020 due to COVID-19 regulations, the interviews were conducted online in the participants’ convenient environment, such as their home or office, with internet connectivity. Before commencing with the interviews, the researcher provided the participant with more information about the study and the structure of the interview session. Pre-determined questions guided the interview:

In your opinion, how effective is your workplace in assisting employees in the changing world of work?
How do you view your role as an industrial psychology practitioner in the changing world of work?

The qualitative survey was completed by participants who had already completed an interview, and from whom additional data was needed. The participants completed their answers in a typed format. The survey questions were as follows:

In your opinion, what is the impact of COVID-19 on the workplace?
In your experience, what is the psychological impact of COVID-19 on employees?
How do you view your role as a workplace counselor to manage the impact of COVID-19 in the workplace?

### Data analysis

Deductive and inductive thematic analyses were utilized to analyze the data. Mayring (2022) indicates deductive thematic analysis as the process of data coding where new information is found in data while referring to previous research findings. The research objectives were used to form a structure of categories where the themes were categorized. Inductive thematic analysis entails working with the participants’ experiences and deriving themes and codes from the data (Azungah and Kasmad, 2018; Mayring, 2022). Since new experiences were recorded from the interviews and surveys, new themes were discovered in the data. To maintain the data’s quality and trustworthiness, the researchers ensured that questions related to the topic were asked and reported an accurate reflection of the participants’ experiences. A qualitative survey was included as the study developed to access more data. Using the survey data, a deductive thematic analysis was followed guided by the research objectives to form a structure of categories where the themes were categorized.

## Results

The study’s results were grouped into three categories, consisting of themes and substantiated by responses from the participants.

### Category 1: The changing world of work

The first category was formed based on the first objective of the study, exploring the role of the industrial psychology practitioner in the changing world of work. Firstly, the participants were asked how effective they view their workplace in assisting employees in the changing world of work. From the responses, the themes were grouped and are reported below.

### Theme 1: Accelerated change

The data shows that from the participants’ perspective, change is constantly present in the current workplace and even accelerated by the onset of the COVID-19 pandemic. The participants indicated that the effect of the changing world of work on employees is increased work pressure and job demands.

*I think it makes work at (sic) faster pace, technology definitely puts pressure on the employees because is the emphasis to learn new systems, new technology, new social media almost on a weekly and monthly basis. There is a lot of change coming in, with change put a lot of stress on employees and it can also cause burnout. (Participant 2, Female).*

The participants indicated that job demands increased in the new world of work thereby putting more pressure on employees. More work pressure results in elevated stress levels and even burnout among employees.

*The impact on employees—the stress level is so much higher, lack of authentic emotional expression and the demands on them have increased. (Participant 4, Female).*

The participants highlighted that employees need to ensure that their skills stay relevant and that they upskill to keep up with the changes the new world of work brings.

…*if they do not upskill, they will become redundant and obsolete. So, obsolescence is one of those things that the employee will face if they are not upskilling or aligning their skill sets to the new challenges in the world of work. People that struggle to adjust will find it very difficult to stay relevant in the new world of work. (Participant 13, Male).*

The results show that different generations react in different ways to the changing world of work. The younger generation seems to adapt faster and cope better than the older generation. The participants further felt that the older generations must upskill to a large extent, especially concerning technological changes.

*Different generations are handling this differently. I think for our millennials or Gen Y are adapting much faster because job security is not that important for them as the older generation*…. *the older generation we are not that responsive to change, and we like to have job security. (Participant 3, Female).*

*I think the challenge we are going to face with the fourth industrial revolution is different between these generations (workforce). Your Millennials will be more inclined to the 4IR because they grew up with using technology and the baby boomers might find it difficult to adjust to it. When 4IR changes are introduced most of the baby boomers, and generation x (older employees) struggle with using it. (Participant 19, Male).*

The data showed that many jobs are affected by the accelerated changes characterized by the new world of work. Some participants indicated that the effect of the changing world of work on employees benefits them by making things simpler.

…*it makes life easier, I think it making life easier, if you look at stuff like AI within the field that I currently work, it is used to help with comparing documents and dealing with different legal areas etc. (Participant 22, Female).*

The participants indicated that the changes presented by the changing world of work, save time and energy as new ways of doing things are faster and easier, and information is easily accessible. The results further showed that the changing world of work is benefiting organizations in that organizations get the opportunity to adapt and implement effective and efficient ways of work.

…*it is relating to digitalisation things, especially from the HR perspective and most importantly with the environment we find ourselves in, is ever changing. Making sure that everything is done in effective and efficient manner in order for the organisation to achieve its success or stay relevant in their specific industry. (Participant 8, Female).*

### Theme 2: Virtual connections

The data showed that in the participants’ experience the changing world of work was introduced by the onset of the 4th industrial revolution. In their experience the new world of work is primarily highlighted by making global connections possible through meetings, conferences and virtual office work for employees.

…*4IR is here, it is not coming. It is not an event; however, it is something that is happening gradually. People are running conference online; you can post something online, and it has global impact. (Participant 11, Female).*

…*the 4IR will change the way we work, and it is going to have an impact on every kind of career. Moreover, organisations that want to stay productive or profitable and want to compete in the global market*…(*Participant 13, Male*).

The participants also indicated that the new world of work had enabled data integration to enable HR data analysis within organizations. Also, the geographic shift that took place in the world of work allows employees to work more efficiently while working remotely.

*Integration of more technology into the workplace, more focused on big data and people analytics not doing big data and people analytics the traditional way (spreadsheet individually). The increased use of technology and using technology in your work processes. We are already functioning in the 4IR and it is evident in the workplace. (Participant 7, Female).*

…*but technology has enabled us to do work remotely and more efficiently because if I am home, I am not spending a lot of time chatting to my friends in the office; therefore, I am actually doing much more with my time when I am working from home. It saves time and it is convenient*… *(Participant 17, Female).*

Next, category two is discussed. The category was formed based on the first objective of the study, exploring the role of the industrial psychology practitioner in the changing world of work. The participants were asked how they view their role as industrial psychology practitioners in the changing world of work.

### Category 2: The roles of the industrial psychology practitioner in the changing world of work

#### Theme 1: Facilitate coping

The participants indicated that their role as industrial psychology practitioners in the changing world of work was one of being a facilitator of the change process within the organization.

*We have a number of responsibilities; the first thing is that we need to serve as change agents, reason being that a lot of jobs are going to have to undergo a large transition in order to remain relevant and some jobs are not going to be relevant. As IOP practitioners we have to serve as stewards for this change for these types of jobs that are coming. (Participant 6, Male).*

*Also making sure that you make it fun like running competitions, changing performance enhancement approach to suit the workforce, and following more contemporary approach. I recurrently lodged the project called growth for growth and the purpose of this project is to build high performing team in the sense that people are able to achieve goals, work collaboratively. (Participant 8, Female).*

The participants indicated that as facilitators of change, they support and guide employees to cope with organizational change initiatives through providing group support such as peer support groups.

*Help people understand 4IR and the interventions the organisation is proposing in response to the 4IR. To ensure that there is an integration between the automated process and the people. By looking at the risk involved in the process the organisation wants to implement and the reason thereof (efficiency, better profit margins, for reduction of Labour force or reduction in the production time) and what is the purpose of implementing these things. Use tools (evidence-based approaches) to understand the impact of the 4IR and address the identified impact. Help organisations to be transparent to employees and manage the relationships (e.g., union). (Participant 7, Female).*

*The role of personal resources has been reaffirmed, and I believe it is part of the role of the IOP to pro-actively invest in resource building, whether it be through training, counselling, coaching, peer support groups, etc. (Participant 13, Female).*

A significant part of the data revealed that the practitioners’ role is to provide support to management in various ways such as providing training and help building relationships.

*I had to assist managers in making adjustments to support staff in order to recuperate which led to me having to look at new ways of applying the 3-intervention levels (individual, team and organisation) in this context. (Participant 5, Female).*

*Line managers equipped to create psychological safety;—line managers’ ability to use 1 on 1 sessions to check on employees and provide adequate support;—leaders modelling appropriate healthy behaviours. (Participant 11, Female).*

#### Theme 2: Helping

A significant role reflected from the data was that of supporting employees through counseling and coaching. The participants shared various responses relating to how support is provided to employees.

*The role of Industrial psychology practitioner is to provide guidance, coaching and counselling. IOP should do a needs analysis of what people are struggling with in terms of the 4IR; therefore, counsel them accordingly. (Participant 20, Female).*

*I think with this industrial revolution (4IR) change is much faster than we have known it to be, and I think my role will be more of supportive role to people to adapt to changes and also to loss. I think in many cases there will be losses and then helping clients and/or employees to craft their own way of doing things and also to assist them that if they experience job loss to go on to a new career path. And helping people to cope with change. (Participant 1, Female).*

By means of using coaching skills the participants felt they could provide a supportive role to employees and assist them with coping with the changes the new world of work brings.

*My role will be to guidance (sic) this person through coaching. The more technology will increase the more and more they will be neglecting of people side. We (IOPs) will have to work harder to ensure that people are well looked after, they know how to balance (work and life), that they know which careers they can choose (obviously the whole career mapping will change going forward, in terms of the impact of technology on the careers). I think in the end our role will have to adapt as Industrial Psychologists providing more support to people and things impacting our workforce in the workplace. (Participant 5, Female).*

The participants indicated that as a behavior-specialist, their role includes understanding behavior and what is needed to support people. The participants reflected on designing tailored interventions based on accurate diagnoses of what is needed, either on individual, group or organizational level.

*In the 4IR as things are becoming more complex and challenging, people are having multiple roles; it is not necessary that you come to work and do a single role. It is going to be having multiple portfolios, stretching your skills, having side-hustle, and having short-term contracts; not having long-term commitments. There is a lot of demands on the individual and for an individual to catch up with those requirements, requires a special touch that is where is see the Industrial Psychologists ideal role is to be able to support the individuals in those transitions. Being able to understand how you can handle that complexity, the dynamics between now that it is like a lot of challenges being faced, is the softer side of things and the harder thing to explain to people in terms of the value. (Participant 11, Female).*

The participants indicated that they view their role within organizations as being responsible for managing employees’ mental health and considering their psychological wellbeing. This was reflected throughout the data gathering process, such as participants 1 and 2, respectively indicated that the workplace could be more effective when they assist employees, as indicated in their extracts below:

*By assisting employees who show signs of trauma or emotional stress with counselling.*

*Supportive role, listening to stories of experiences, exploring career changes with clients and new self-awareness that came with the pandemic. Emotion management and regulation re-building relationships at work, exploring and healing and coping with this traumatic event.*

#### Theme 3: Gatekeeper

Lastly, the participants further indicated that their role within the organization is to highlight transparency and be stewards of ethical change management. As a gatekeeper, this included ensuring that data is managed responsibly and that considerations regarding implementing the changes flowing from the changing world of work are fair and ethical.

*The biggest role is ethics. I think we need to be careful around help manage and control companies around how to capture and keep data. Because there is a lot of potential for the data to be misused and manipulated in a way*…*. (Participant 6, Male).*

*I always think our role is a bit of a check and a balance, ethical viewpoint. So, we will always want people to come to us and ask is that right, ethical and kind, all the considerations/decision around people. We also want to be looking not just making sure things are fair, safe, kind, and ethical sound for people, but also in terms of enhancing the wellness of people. For it is the ethical foundation and making sure that things are done properly, and people are being considered because things are going fast it is easy to forget the people side as to how we ensure that people are being enhanced. (Participant 11, Female).*

Next, category three is discussed, which relates to the second objective of the study, exploring the role of the industrial psychology practitioner as a workplace counselor to manage the *impact* of COVID-19 in the workplace.

### Category 3: Managing the impact of COVID-19 in the workplace

Category 3 was formed when the participants were asked their opinion on the impact of COVID-19 in the workplace and the psychological impact of COVID-19 on employees. Four themes emerged from the data and are reported next.

#### Theme 1: Culture change

The participants indicated that the impact of COVID-19 in the workplace is evident through culture changes that took place in organizations. They reflected on how the new normal included working from home, managing a virtual office, learning new work-life balance dynamics and managing employees differently.

*Impact of COVID-19 is both positive and negative. Positive; in challenging our old ways of thinking - Covid-19 opened up doors to hybrid-working cultures securing a better work life balance. The impact has also challenged individuals and companies to be more agile and adaptable. (Participant 3, Female).*

*The workplace moved from a physical office to a digital environment. Employees had to work from their homes and share the limited space with family members. (Participant 8, Male).*

The participants indicated that the way life and work were before the pandemic had forever changed. In a positive sense, the participants felt excited about opportunities for hybrid working and a more balanced way of living. Employees working from home seemed more relaxed in this flexible environment. Furthermore, participants also indicated that organizations must relook work practices, such as providing a safe workplace within the context of COVID-19 (facilities and training staff on safety protocols).

… *adoption of work from home at a massive scale, balancing living in a pandemic and all the new global and country regulations while trying to be productive, opportunities for companies to consider work arrangements and supporting managers to manage remotely. (Participant 9, Female).*

*There is more flexibility from managers who used to be clock watchers. Managers now realise that employees can work from home if they need to take care of the kids etc. (Participant 1, Female).*

#### Theme 2: Mental health

In addition, the participants indicated that a significant part of the impact of COVID-19 was on employees’ mental health. Apart from the effect on employee health, employees had to adapt to many changes, some employees showed signs and symptoms of ill health, while others showed resiliency and engagement.

*At the start of the pandemic employees felt overwhelmed and anxious. Many had to dig deep to find resilience to be agile in the changing environment. As people worked through the changes in their different manners, the psychological impact ranged from negative (i.e., depression) to positive (i.e., sense of meaningfulness). However, as the pandemic continued my sense was that most people’s personal resources were running low and that they experienced symptoms of burnout. As each individual’s experience is so unique, the pandemic has taught us to heighten the focus on mental health in the workplace, and to provide individualised responses. Now in 2022 the culture of overwork and unreasonable expectations are surfacing again. (Participant 10, Female)*.

The results showed that employees who fell ill and lost loved ones due to COVID-19 were especially at risk of showing PTSD symptoms. The participants reflected on ways of addressing signs of burnout, depression, trauma and PTSD in employees.

*In my consultations I have noted that employees are still extremely fatigued even after 3 months of contracting Covid. I notice an increase in reports of burnout, and depression of employees who were recovering from Covid. People who lost someone due to Covid are also left traumatised. Those who had Covid and those who didn’t were equally fearful of contracting the disease which led to increased levels of stress. Working from home had a good effect on some but a negative effect on others. (Participant 13, Female).*

*Covid has caused a lot of trauma for employees where some have lost a loved one or there was a financial impact on the family. In my opinion this is causing employees to be less resilient towards the normal stresses in life. (Participant 1, Female).*

The participants indicated that in their experience, the impact of COVID-19 in the workplace included job loss and uncertainty, which also led to mental health problems such as anxiety and depression.

*Due to loss people experience some degree of depression, due to being isolated some experience a degree of anxiety as well as depression, and then there’s individuals that experience prolonged Covid which impacts them mental and physically. (Participant 7, Female).*

The participants stated that they know of many companies that had to close their doors, lost or digitized jobs, and employees had to take salary cuts. Participant 9 supported this by writing in the survey: “*Increased pressures on mental health overall on individuals; massive job losses and increased anxiety on job security and balancing the grey area between work and home and “always-on” culture.”*

The participants felt that as employees faced much loss, levels of fear also increased. One participant explained that employees experienced high levels of stress due to many different reasons such as stressors of financial strain, uncertainty about the future, concern about the possibility of infection, and travel restrictions. The participants indicated that employees felt fatigued, and a high prevalence of burnout was noted among employees returning to work.

*Trauma of leaving workplace suddenly in March 2020 caused post traumatic stress. Anxiety of not knowing how the pandemic will play out. Information overload and bombardment of training availability and expectations to learn new methods in a short period increased stress levels. Emotional numbness now in learning anything new. Fear of other changes still too come. Similar to surviving a war but still exited to perform on a very high level. Returning to work very, very challenging, yet another change. (Participant 2, Female).*

*I think it has increased fear in general, each person’s trigger around fear was pressed. Whether it was fear around income, job security, health etc. I think as a summary it removed peoples sense of certainty and increased discomfort, which has a knock on effect on all our triggers. (Participant 8, Male).*

#### Theme 3: Availability of resources

The participants further indicated that the significant impact of COVID-19 in the workplace was how it impacted the availability of resources. As such, organizations are faced with the change of adjusting and adapting at a fast pace and should ensure the efficient availability of resources exist.

*Unprecedented, this has not been seen since the Spanish flu. The workplace has to adjust and adapt at lightning speed and also figure out how to support employees who were left traumatised by the pandemic. (Participant 13, Female).*

In addition, participants indicated that the impact of the pandemic could also be felt by technological advances, as employees worldwide were requested to work from home. Some employees were confronted with the challenge of not having an internet connection to be able to work effectively.

*Many employees did not have adequate connectivity or data to continue effectively with their duties. Employees had to upskill very quickly regarding digital and technological capabilities. Video conferencing platforms such as Zoom and MS Teams became the mode of communication and collaboration and employees had to adjust quickly and learn how to utilise these digital applications. (Participant 8, Male).*

The participants indicated that working remotely had its benefits, but post-pandemic, it is clear that employees have to relearn how to function in a team and socialize as a group again. The availability of peer support as a resource is evident as Participant 1 state:

*Teams have had to learn to work together again in a face-to-face environment. While working from home I had limited contact with my colleagues, now I am back at the office, and I have to face them for 8 hours a day. People need to learn how to again accept and cope with other members of the team who they don’t necessarily get along with. Social traditions that were established before Covid has gone and effort needs to be put in to reinvent these traditions (socials, drinks after work etc.).*

#### Theme 4: Meaningfulness

The participants reflected that the sense of loss they observed from employees were also seen when companies requested their employees to return to the office, and employees now faced a sense of loss of the security they had while working from home. The participants indicated that, in their opinion, the impact of COVID-19 in the workplace is that as employees searched for meaning in their experiences, many employees report having good experiences as they re-evaluated what mattered in life. A prominent shift was reported in resignations as people explored other areas of life to “find more joy” (Participant 6). The data showed that most participants indicated that the onset of COVID-19 led to some employees living a more purposeful life.

*It shook everyone and made them clarify what they want from their life, what is important and this has to lead to people feeling niggly in wanting to make changes, find more joy, move closer to a life they want. I believe people are trying to cope and are also stretching for more. (Participant 6, Female).*

## Discussion

The results indicated that the new world of work’s influence on organizations was prevalent in the rapid advancement of technology in recent years. This was especially evident from how knowledge and information were distributed and how jobs were affected, thereby accelerating change, and automation and digitalization in organizations. Mayer and Oosthuizen (2021) found in their study among managers in a South African technology organization that the challenges of the 4IR highlighted both the positive and negative experiences of the participants regarding the rapid and disruptive changes within the organizations. According to the participants the transition to the new world of work dramatically impacts work. The manner in which work is being done, having less human interaction, and simplifying organizations are a few examples the participants provided of the impact of the transition. Similarly, Schwab and Davis (2018) revealed in their study that the changing world of work is reshaping how work is performed. The participants indicated that they found that different generations coped differently with this transition. While the younger generation seemed to find it easier to transition to the new world of work, the older generation felt a sense of job insecurity because of automation and digitalization in the workplace, which forced them to upskill and learn to adapt to the changes. Mariano et al. (2021) report that older employees are often stereotyped as less technologically skilled than younger employees and thus tend to avoid using technology.

The study results also showed that the participants felt that apart from the changes employees’ experience in the workplace due to the new world of work, the COVID-19 pandemic brought about a further significant culture change in the workplace. The pace of work accelerated and the constant change in the workplace evolved to a next level since the pandemic started. The results show that the participants noted that the culture shift in the workplace was related to the new way of work, including working more remotely, managing a virtual office, work-life integration, and a feeling of constant change. The participants reflected on the prevalence of depression and a sense of loss at the pandemic’s beginning. This was possibly intensified by the isolation of lockdown, loss of loved ones as well as the threat to the employees’ health. The participants in the study observed a strong sense of job uncertainty and insecurity among employees they supported. The participants indicated that some organizations had to close, jobs were redundant, and some jobs changed. The findings are in accordance with the study of Giorgi et al. (2020), who found that social distancing policies, the lockdown, loss of income and fear of the future led to an influence on the mental health of employees.

The results of our study show that the participants identified signs and symptoms of psychological trauma due to the COVID-19 pandemic among employees. The shock of leaving work and life as it was known so suddenly, followed by the threat of falling ill and the death of loved ones, led to a prevalence of trauma symptoms among employees. Restauri and Sheridan (2020) state that the description of the COVID-19 pandemic compared to a traumatic event as defined in the Diagnostic and Statistical Manual of Mental Disorders (American Psychiatric Association, 2013). A traumatic event is more severe than a crisis and has a more unpredictable onset. Literature states that trauma involves an individual experiencing, witnessing or being confronted with actual or threatened death, serious injury, or threat to others or the self (American Psychiatric Association, 2013). An individual’s response to the trauma includes feeling intense helplessness or horror. This definition accurately describes most people’s experiences during the pandemic. Exposure to a traumatic event could lead to the onset of acute stress disorder and if symptoms continue to persist, PTSD (Restauri and Sheridan, 2020). The vulnerability of South Africans to post-traumatic stress disorder is highlighted by Naidu (2020) due to the collective trauma of the past.

As employees return to work, the participants in this study indicated that they noticed a culture of overwork and unreasonable expectations of employers. Ingusci et al. (2021) state that workers are required to work under pressure for longer hours with increased workloads. It is essential for employers to, especially during the pandemic, review tasks and have realistic expectations (Ingusci et al., 2021). Post-pandemic management involves integration back to a work environment that has changed drastically (Boissé, 2021). Employees need to be supported to re-establish routines and to support anxious employees. Roberge (2020) states that management should be vigilant in supporting employees returning to work. This can be done in three stages, first focusing on the human aspect, ensuring that employees are coping, emotionally well and thriving. Secondly, in a transition period, establish more contact points during the day to check on employees as they return to a more normal routine, either at work or home. These could include enquiring if employees have what they need (resources), checking on how employees are coping and enquiring whether they are taking enough breaks. Lastly, focusing on the business aspect of an organization, management should ensure that the job is being done, and that adjustments to task agreements are made to accommodate new work as the world of work changes. Our study showed that employees need support from management in order to cope with the new world of work. More so after the pandemic as employees need to adjust to how the world of work changed.

While the results show that COVID-19 mostly had a negative effect on work-life, some participants reflected on how they noticed a sense of meaningfulness in some of the employees they supported. The participants noted that employees seemed to revisit what is important for them in life, reshaping their work and family lives and reconnecting to what is important. An American organizational psychologist, Anthony Klotz, stated in an interview with Bloomberg that many employees are rethinking where and why they want to work (Cohen, 2021). Employees are re-evaluating meaning in terms of family time, remote work, finding joy in projects and meaning in life (Cohen, 2021). Employees can consider their choices (Smith, 2022), which led to many employees resigning and seeking other job opportunities. Much has been published about the “mass resignation” phenomenon worldwide as employees seek employment elsewhere, among others, seeking remote work opportunities (Iacurci, 2022). This involves more than resigning from a job, but also involves individuals taking control of their work and personal lives (Cohen, 2021).

The participants indicated that they felt their role prior to the COVID-19 pandemic revolved around assisting employees in crafting new career opportunities or new work arrangements, and new ways of doing work. This role has intensified significantly since the COVID-19 pandemic started. In their report, Deloitte (Volini et al., 2021) states that in the post-pandemic workplace, work should be re-defined. The report indicates that a focus should be placed on the “art of the possible” for what can be achieved using technology, thereby enabling and elevating human capabilities. Our study showed that the participants believed that organizations are exploring ways to manage employees who work remotely permanently related to managing outputs and not merely managing the hours during a working day. Increasingly, more studies show that organizations seek to attract and retain talent by providing employees autonomy over their work schedules (Cohen, 2021). More flexibility at work could lead to improved mental health and better work-life balance (Cohen, 2021).

Some participants reflected on how coming through the pandemic was like surviving a war, but they were still excited to perform at work. In recent years, publications have reported how people recover from adversity (Ryff et al., 2012), and how this positive recovery from adversity can be described as resiliency. Ryff et al. (2012) define resilience as “the maintenance, recovery, or improvement in mental or physical health following challenge” (p. 794). It is possible that the responses from the participants in our study could reflect on the post-traumatic growth theory (Tedeschi and Calhoun, 2004). The theory states that people who endure psychological struggles following adversity often see positive growth afterward.

Facilitating growth and development among employees is a significant part of industrial psychology. Industrial and organizational psychologists are ideally suited in organizations to support employees in the current post-pandemic phase by following an intervention approach suited for employees in their specific trajectory. A good example is Banerjee and Nair’s (2020) intervention toolkit, developed to support facilitators dealing with employees’ mental health during a pandemic. The approach to psycho-social interventions speaks to the industrial psychology professions and involves needs assessment, crisis management, open communication, individual and group support and interventions, and organizational restructuring. Nguse and Wassenaar (2021) state that the role of psychologists during the COVID-19 pandemic includes being at the forefront of this task and sharing resources to assist with mental health issues in the community in South Africa. Training psychologists and mental health professionals in immediate and responsive treatment measures such as psychological first aid, solution-focused brief psychotherapy and longer-lasting treatment models are vital to prepare for similar mental health challenges such as the COVID-19 pandemic (Graupner, 2021; Nguse and Wassenaar, 2021). van Lill and van Lill (2022) recommend training in the Acceptance and Commitment Therapy (ACT) for Work Well-being model, particularly suitable for industrial psychologists who offer brief counseling interventions. The participants reported that their role in the changing world of work is to facilitate support such as providing services such as counseling and coaching. In this regard, the industrial psychology practitioner is concerned with talent management with organizations; in their capacity as counselors, coaches and people-developers, they equip individuals to address their personal or developmental needs (Jorgensen et al., 2016; Van Zyl and Stander, 2016). The industrial psychology practitioners in this study reported that their role as workplace counselors involves supporting employees to adjust to the workplace, especially during the pandemic. To manage wellbeing in the workplace, the industrial psychologist should be concerned with supporting employees’ adjustment (Health Professions Council of South Africa [HPCSA], 2019), more so in the post-pandemic phase. van Lill and van Lill (2022) indicate that it can be considered an obligation that industrial psychologists have to their organizations and an ethical duty as psychologists to intervene when employees present with mental health challenges.

The significant role of the industrial psychology practitioner in managing the impact of COVID-19 in the workplace became quite clear from the participants’ responses. The participants indicated that an important role of industrial psychology practitioners in the changing world of work is being ethics managers. Participants reported that as ethics managers, they were responsible for managing ethics by gatekeeping in organizations, ensuring that the organizations adhere to legislation such as the Protection of Personal Information Act (POPI Act) in South Africa. The results also indicated that the participants felt it is their responsibility to keep the workplace fair and safe. Jorgensen-Graupner and Van Zyl (2019) assert that positive ethical behavior is a foundation of industrial psychology practitioners. In this regard, Van Zyl et al. (2016) confirm that industrial psychology practitioners maintain ethical standards and practices within organizations. It would therefore seem to implicate that industrial psychology practitioners should assist managers in making the workplace a safe space for employees to return to work after the COVID-19 pandemic or to assist in rethinking how the new workspace should be managed as a safe environment.

It is recommended that organizations invest in proactive and reactive interventions to address the employees’ mental health. Organizations should focus on managing job demands and evaluating job support since new challenges emerged during the COVID-19 pandemic regarding workload. A stronger focus on the accessibility of interventions on virtual platforms is immanent for access to employees working from home. A focused effort in training line managers in identifying signs and symptoms of psychological ill-health among staff members is necessary to provide adequate mental health support. Industrial psychology practitioners could use the findings of this study to position themselves as change agents, consultants to management, ethics managers, and workplace counselors to adequately facilitate support in organizations to manage the psychological impact of COVID-19. Industrial psychology practitioners must continuously develop counseling skills to facilitate psychological support to employees. In hindsight, it is clear that training in crisis management skills should be awarded priority to manage the psychological impact of the changing world of work, and also traumatic incidents such as the COVID-19 pandemic, in the workplace.

## Conclusion

In conclusion, the findings in the study show that industrial psychology practitioners have a significant role in supporting employees through traumatic and stressful incidents, such as the COVID-19 pandemic, using counseling theory and models. It is recommended that industrial psychologists focus on continuously developing their skills in individual, group and organizational counseling interventions to be ready to support employees optimally.

## Data availability statement

The raw data supporting the conclusions of this article will be made available by the authors, without undue reservation.

## Ethics statement

The studies involving human participants were reviewed and approved by the Faculty of Economic and Management Sciences Ethics Committee (NWU), North-West University. The patients/participants provided their written informed consent to participate in this study.

## Author contributions

TM conducted this study as part of his master’s dissertation. He was responsible for the data collection, data interpretation, and writing of the dissertation and manuscript for publication purposes. LG supervised the study, helped to conceptualize the study, assisted with data analyses, data interpretation, and the writing up of the manuscript for publication purposes. Both authors contributed to the article and approved the submitted version.
